# A Survey of Diving Behavior and Accidental Water Ingestion among Dutch Occupational and Sport Divers to Assess the Risk of Infection with Waterborne Pathogenic Microorganisms

**DOI:** 10.1289/ehp.8523

**Published:** 2006-02-16

**Authors:** Jack Schijven, Ana Maria de Roda Husman

**Affiliations:** Microbiological Laboratory for Health Protection, National Institute of Public Health and the Environment, Bilthoven, the Netherlands

**Keywords:** *Campylobacter*, divers, enteroviruses, risk of infection, volume of water

## Abstract

Divers may run a higher risk of infection with waterborne pathogens than bathers because of more frequent and intense contact with water that may not comply with microbiologic water quality standards for bathing water. In this study we aimed to estimate the volume of water swallowed during diving as a key factor for infection risk assessment associated with diving. Using questionnaires, occupational and sport divers in the Netherlands were asked about number of dives, volume of swallowed water, and health complaints (nausea, vomiting, diarrhea, and ear, skin, eye, and respiratory complaints). Occupational divers, on average, swallowed 9.8 mL marine water and 5.7 mL fresh surface water per dive. Sport divers swallowed, on average, 9.0 mL marine water; 13 mL fresh recreational water; 3.2 mL river, canal, or city canal water; and 20 mL water in circulation pools. Divers swallowed less water when wearing a full face mask instead of an ordinary diving mask and even less when wearing a diving helmet. A full face mask or a diving helmet is recommended when diving in fecally contaminated water. From the volumes of swallowed water and concentrations of pathogens in fecally contaminated water, we estimated the infection risks per dive and per year to be as high as a few to up to tens of percents. This may explain why only 20% of the divers reported having none of the inquired health complaints within a period of 1 year. It is highly recommended that divers be informed about fecal contamination of the diving water.

Exposure to waterborne pathogens in surface water may lead to health complaints among recreants such as bathers, divers, surfers, kayakers, and anglers. In the Netherlands, because of discharges of raw and treated sewage and manure runoff from agricultural land, pathogenic microorganisms may enter surface waters (e.g., Lodder and de Roda Husman 2005; Schijven et al. 2004; Van den Berg et al. 2005). Swallowing this water may lead to infection, which may lead to symptoms such as nausea, fever, and diarrhea or more severe illness. *Campylobacter* species and waterborne viruses are of major importance in that respect (de Roda Husman 2001; Schijven 2003). In addition, there are pathogens indigenous to surface water, such as *Pseudomonas aeruginosa*, *Vibrio*, amoebae, and cyanobacteria. In the Netherlands, skin complaints, followed by gastrointestinal complaints, were reported most often among water recreants (Leenen and de Roda Husman 2004; Schets and de Roda Husman 2004). Skin complaints were especially ascribed to cercaries and cyanobacteria. In 1994 and 1995, ear complaints caused by *P. aeruginosa* (otitis externa) were important incidents in the Netherlands involving large numbers of patients (Van Asperen etal. 1995).

Water-associated health complaints may occur despite the fact that the microbiologic quality of the bathing water complies with European Union (EU) Directive 76/160/EEC (1976), which sets limits for fecal indicator bacteria and is primarily aimed at protecting the bather against gastrointestinal complaints and acute febrile respiratory illness, but not against eye, skin, and ear complaints. Epidemiologic studies have demonstrated that these legal standards protect bathers insufficiently (Kay et al. 1994; Van Asperen et al. 1998; Wiedenmann et al. 2006). The proposed new EU bathing water directive COD 2002/0254 (2002) aims to better inform the water recreants on the risks of bathing by means of bathing water profiles. This proposed directive also addresses the fact that water activities other than bathing, such as diving, surfing, and kayaking, have strongly developed since 1976. Divers, surfers, and kayakers may be exposed to a greater extent to waterborne pathogens than are bathers because of more often and longer contact with surface water that need not be recreational water and that may be more fecally contaminated than are recreational waters. Therefore, persons that practice these activities may well be subjected to a higher health risk than are bathers. Several studies have reported health problems related to water activities other than bathing. Alcock (1977) reported outbreaks of otitis externa caused by *P. aeruginosa* in saturation dives in the North Sea. During saturation dives, the diver’s tissue gasses reach equilibrium with the aqueous environment, allowing near unlimited time working underwater. Occupational saturation divers may acquire various skin disorders, of which skin infections, most often caused by *P. aeruginosa*, are the most serious and frequent (Ahlén et al. 2003). Skin lesions and skin infections provide opportunities for microorganisms and toxic chemicals to penetrate under conditions of hydrostatic pressure (Richter et al. 2003). Losonsky et al. (1994) found a significant increase in the frequency of isolation of *Pseudomonas* and *Aeromonas* from respiratory surfaces and predominantly the divers’ ears. The rates of seroresponse to these microorganisms suggested that repeated exposure is necessary for generation of a specific systemic immunologic response and that there are various levels of susceptibility to waterborne pathogens in both experienced and inexperienced divers. Garin et al. (1994) found a higher percentage of divers seropositive for coxsackievirus B4 and B5 compared with a control group. Dewailly et al. (1986) documented the risks associated with windsurfing on sewage-polluted water. Relative risks were 2.9 [95% confidence interval (CI), 1.3–6.6] for occurrence of one or more symptoms of gastroenteritis, otitis, conjunctivitis, and skin infection and 5.5 (95% CI, 1.4–21.4) for symptoms of gastroenteritis only. Relative risk increased with the reported number of falls into the water.

A key factor in determining health risks involved in exposure to pathogens in surface water is the volume of water that is being swallowed. To date, no study has aimed to estimate volumes of swallowed water. Kay et al. (1994) related relative incidences of gastroenteritis with concentrations of fecal indicator organisms and head immersions of swimmers. Similarly, Wiedenmann et al. (2006) related relative incidence of gastroenteritis with head immersions of swimmers and whether the swimmers had swallowed water in a single period of 10 min. The mean risk attributable to swallowing water above threshold concentrations was significantly higher (3.6%) than the attributable risk below threshold concentrations (1.3%). Neither of these studies quantified volumes of swallowed water; instead, head immersions were looked upon as an equivalent for the uptake of water.

In the present study we aimed to collect data on the volume of water that is swallowed during diving. Toward that aim, such data were collected by means of questionnaires sent to occupational and sport divers in the Netherlands. Divers are an interesting group to study because diving involves full immersion in the water for a relatively long period of time, allowing maximum exposure on the one hand, but on the other hand, divers immerse in a very controlled manner as opposed to accidental immersions of swimmers, surfers, and kayakers. In addition, we collected literature data on concentrations of *Campylobacter jejuni* and enteroviruses in surface waters, for example, and calculated the risks of infection with these pathogens. Furthermore, divers were asked about health complaints that could have been caused by infections acquired during diving.

## Materials and Methods

### Questionnaires.

We used questionnaires to ask occupational and sport divers about the number and duration of dives for various types of surface waters, the amount of water that was swallowed per dive, and the type of diving mask that was worn. Also, questions were included about health complaints that may possibly have been due to an infection from a waterborne pathogen. The questionnaire for the occupational divers concerned the year 2002, and that for the sport divers was for 2003. The questionnaire for the occupational divers was constructed in consultation with the Dutch Association of Diving Enterprises (NADO) and was sent by letter in February 2003 to 25 Dutch diving enterprises with 233 occupational divers. NADO represents 95% of the Dutch diving industry with about 500 employed occupational divers (1–30 divers per enterprise). The questionnaire for the sport divers was constructed in consultation with the Dutch Divers Union (NOB) and by using the experience that was obtained from the questionnaire for the occupational divers. NOB has approximately 26,000 members. The questionnaire was announced to the sport divers by means of the NOB journal *Onderwatersport* and on their website (NOB 2006). The questionnaire was made accessible from January through April 2004 as an Internet form on the website of the National Institute for Public Health and the Environment (RIVM 2006) and was linked to the NOB website. Sport divers were required to identify themselves by means of their membership code.

Types of occupational divers were starter, second diver, first diver, all-round diver, and team leader. Sport diver types were diver with no certificate, classes 1*–4*, and instructor. The types of water for occupational divers were open sea and coastal and fresh water. In addition, a distinction was made for the presence of sewage discharge within 1 km upstream. In the case of sport divers, the types of water were open sea, coastal water, fresh recreational water, canals and rivers, city canals, and swimming pools. In the case of sport divers, no further subdivision by the presence of sewage discharge was made.

Occupational divers commonly wear a full face mask or a diving helmet and sometimes scuba gear with an ordinary diving mask. Sport divers always wear an ordinary diving mask and sometimes a full face mask. The divers were asked to estimate how much water they swallowed in terms of a few drops of water up to a soda glass full (Table 1). Questions about health complaints encompassed respiratory, eye, skin, and ear complaints; diarrhea; vomiting; and nausea.

### Data analysis.

The data from the questionnaires were scored using Mathematica (version 5.0; Wolfram Research Inc., Champaign, IL, USA). Subsets of data were constructed according to sex, certificate, type of diver, diving mask, and water for analysis of variance (ANOVA). For comparison, also data on sex, age, length, and weight for the Dutch population in 2003 were collected using Statline (Statistics Netherlands 2005).

### Volume of swallowed water.

We translated the descriptive swallowed volumes from the questionnaires into the average volumes (milliliters) as shown in Table 1. Obviously, the duration of a dive is relevant for the exposure assessment; however, we did not take this into account because it was too difficult for a diver to estimate the volume of swallowed water per time unit.

### Number of dives.

In the questionnaires for the occupational divers, divers were asked about the number of dives in 2002 (0, 1–10, 11–20, 21–50, 51–100, 101–200) for each type of diving water. The number of dives was calculated from the group means. The sport divers reported actual numbers of dives for each type of diving water and for each quarter of 2003.

### Risk of infection.

Risks of infection with waterborne pathogens, in this case, *C. jejuni* and enteroviruses as examples, were estimated from the volume of swallowed water and the pathogen concentration. The infection risk per dive, *p_d_*, was calculated using the hypergeometric dose–response model (Teunis and Havelaar 2000):


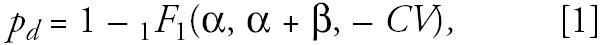


where _1_*F*_1_ is the hypergeometric distribution, α and β are the parameters of the Beta-distribution, *C* is the pathogen concentration, and *V* is volume of water. In the case of *C. jejuni*, the best estimates of parameters α and β are 0.145 and 8.007, respectively, and in the case of enteroviruses, the best estimates of α and β are 0.167 and 0.191, respectively (Teunis and Havelaar 2000). Effects of recurrent exposures either with short- or long-time intervals were not considered. We collected concentration ranges of *C. jejuni* and enteroviruses for certain types of water from the literature (Table 2). Assuming these concentration ranges were the 99% intervals of log-normally distributed pathogen concentrations, we constructed concentration distributions using Monte Carlo sampling (10,000 samples). Table 2 shows the arithmetic mean concentrations for each type of water. Because of their sensitivity to chlorine disinfection, we assumed that both pathogens were absent in swimming pools.

We calculated infection risks per dive, *p_d_*, using Equation 1 with random sampling from the concentration distributions and the swallowed volumes of water for each type of water. Infection risks per year, *p_y_*, were calculated by random sampling from the *p_d_* distributions as many times as the number of dives per diver, *N*, according to


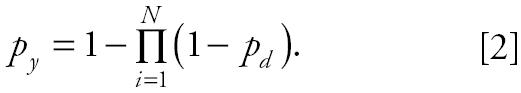


## Results

### Response to the inquiries and general characteristics of the divers.

We received questionnaires from 37 occupational divers from 8 of the 25 enterprises. The response between diving enterprises varied between no response at all to 100% (average, 16%). Only one of the 37 respondents was female. In total, 483 sport divers responded to the inquiry (2.1%), of whom 10% were females (49) and 90% males (433). In 2005, NOB had a total number of members of 26,133, of whom 25% (6,576) were female and 75% (19,577) were male; therefore, female divers appear to be underrepresented in the study. The average age of the divers who took part in this study was very much the same as that of all NOB members and that of the Dutch population. Also, these divers had similar average body lengths and weights as Dutch people >20 years of age.

No apparent differences existed between the certificates of the female and male sport divers and between the sport divers and NOB members, except that no female instructors took part in the study.

### Duration of a dive.

According to ANOVA, no significant differences existed in the duration of a dive between the types of diving water among either the occupational or the sport divers. However, occupational divers dived on average (60–95 min) significantly longer than sport divers (42–52 min). The dive duration of sport divers, who reported per quarter, was relatively constant throughout the year.

### Number of dives.

Table 3 summarizes the percentage of divers by type of water and the number of dives per diver in a year. Two of the 37 occupational divers did not report the number of dives per type of water. For the occupational divers, no distinction is made in Table 3 between wearing an ordinary diving mask, a full mask, or a helmet, because divers wore either of those, usually a full mask or a helmet, for a particular type of water. Taking into account all the possible combinations would divide the divers into groups of only a few divers. For the same reason, no distinction was made between type of occupational diver. Differences in numbers of dives between types of sport divers were not apparent. Differences in numbers of dives between water types were highly significant.

More occupational divers than sport divers dived in open sea. Most of both types of divers dived in coastal and fresh waters, but a few of the sport divers dived in rivers, canals, and city canals. The number of dives in open sea was about the same as that of diving in coastal water when not considering the presence of sewage discharge. Occupational divers dived more often in fresh water than in seawater.

Sport divers dived about four times more often in April–September than in the winter months October–March in all surface waters. In swimming pools, they dived about twice as often in October–March as in April–September and least often in July–September. Although we collected no seasonal data on the number of dives of occupational divers, this number is known to vary little between seasons (Struik PJ, personal communication). Offshore, occupational divers dive throughout the year except under bad weather conditions such as storms. Diving frequencies of sport divers when wearing a full face mask were two orders of magnitude lower. Occupational divers dived more often in surface waters than did sport divers.

### Volume of swallowed water.

Table 1 shows the distributions of the volumes of swallowed water per dive. Most frequently, the divers reported swallowing no water or only a few drops. However, in the case of open sea and coastal water, divers also frequently reported swallowing enough water to fill a shot glass. Higher volumes were reported much less frequently.

Table 3 shows the calculated volume of swallowed water per dive for each type of water. For volumes that were not significantly different according to ANOVA, estimated volumes from combining the data of these types of water are also given. The estimates of the combined data were applied in the infection risk calculations. The occupational divers swallowed about twice as much water per dive in seawater and coastal water than in fresh water. Sport divers wearing ordinary diving masks swallowed as much seawater and coastal waters as did the occupational divers, but occasionally much more. Sport divers swallowed more water when diving in fresh recreational water, but much less in canals, rivers, and city canals. Apparently in the latter cases, divers were more cautious. The highest volumes were swallowed in swimming pools.

One of six occupational divers reported swallowing no water when wearing scuba gear with an ordinary diving mask, 10 of 25 when wearing a full face mask, and 25 of 26 when wearing a diving helmet. Sport divers wearing a full face mask appeared to swallow about 10 times less water per dive than did sport divers wearing an ordinary diving mask. This strongly indicates that much less water was swallowed when divers wore a full face mask instead of an ordinary diving mask and even less when wearing a diving helmet.

### Risk of infection.

Table 4 shows the infection risks per dive, *p_d_*, and per year, *p_y_*. The infection risks for *C. jejuni* are generally one order of magnitude higher than for enteroviruses. This is mainly because of the chosen concentration ranges and the high sensitivity of the infection risk for the concentration values. In the case of *C. jejuni*, the upper limit of the concentration range was always as high as 10^4^ per liter, regardless of the presence of sewage discharge (Table 2). This high limit was chosen because birds may still be present and contaminate the water. A 10-times lower upper concentration limit would lead to an approximately 10-times lower estimate of the infection risk. Also, the 97th percentile of the risks are usually 10 times higher than the arithmetic mean risks.

The mean risk of infection with *C. jejuni* is generally near 1% per dive for occupational divers and sport divers wearing ordinary diving masks. This risk is 10 times lower for sport divers wearing full face masks. The risk of infection with enteroviruses per dive differs more among the water types because of the different concentration ranges of enteroviruses, and varies between 0.02 and 0.3%.

Differences between the annual infection risks are more apparent than between the risks per dive because of the inclusion of the numbers of dives. In the case of occupational divers diving in fresh water with unknown sewage discharge, *p_y_* is highest because of the highest number of dives in that water. Although canals, rivers, and city canals may be more fecally contaminated than fresh recreational waters, the infection risk per year for sport divers was lower for the rivers and canals because they swallowed less water per dive and the number of dives was very low.

### Health complaints.

**Figure 1:**
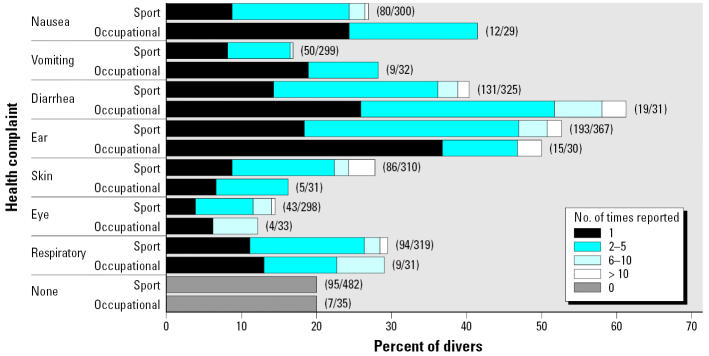
Percentage of divers who had each health complaint of the divers who reported health complaints. Values in parentheses indicate the number of divers who reported that complaint of those who reported health complaints.

Figure 1 shows the percentages of divers who reported health complaints in the study year. For each complaint, about half were reported to have occurred once and about half occurred two to five times. Only 20% of the divers reported having none of these health complaints at all. Occupational divers reported diarrhea very often. Diarrhea, vomiting, and nausea were reported more often by occupational divers than by sport divers, probably because occupational divers dive more often in more heavily fecally contaminated water than do sport divers. According to testing in a 2 × 2 contingency table, occupational divers reported nausea significantly more often than sport divers (*p_x_*_^2^_ = 1%), but the frequencies of reporting the other complaints were not significantly different between occupational and sport divers, partly due to the relatively small number of occupational divers in the study. Both occupational and sport divers reported frequent ear complaints (about 50%). Sport divers reported skin complaints more often than occupational divers. The latter commonly wear fully closed suits, and the former commonly do not. Respiratory and eye complaints were reported as often by both types of divers.

The occupational divers reported having had nausea, vomiting, and diarrhea in January–March and September–December, but not in April–August. On the contrary, they reported having ear complaints only in the summer months. The sport divers reported 40% more ear, skin, and eye complaints; 20% more nausea; and 60% more diarrhea in the warmer months (April–August) when they dive more often, compared with the colder months. They reported 45% more respiratory complaints during the colder months (September–March).

The occupational divers visited a general practitioner for diarrhea (2/35 = 6%), ear complaints (4/35 = 12%), and eye complaints (2/35 = 6%), and the sport divers for nausea (3/482 = 0.6% of cases), vomiting (2/482 = 0.4%), diarrhea (6/482 = 1.2%), ear complaints (98/482 = 20%), skin complaints (30/482 = 6.2%), eye complaints (15/482 = 3.1%), and respiratory complaints (43/482 = 8.9%).

## Discussion

By means of questionnaires, occupational and sport divers were asked to estimate how much water they had swallowed after diving as a key factor for risk assessment. There appeared to be consistence in these estimates, supporting reliability of the data: the estimated volumes of swallowed water for similar types of water could be combined (Table 3). In all cases, uncertainties in the estimated volumes were quite large, with a high frequency of small volumes and a low frequency of high volumes, which is plausible.

We estimated that occupational divers swallowed an arithmetic mean of 9.8 mL and 5.7 mL of water per dive in marine and fresh waters, respectively. There was no difference between the volumes of swallowed water in water with known or unknown sewage discharge, which shows that occupational divers were in most cases not aware of the presence of sewage discharge. Per dive, sport divers swallowed an arithmetic mean of 9.0 mL of water in marine waters; 13 mL in fresh recreational water; 3.2 mL in canals, rivers, and city canals; and 20 mL in swimming pools.

Divers swallowed 10 times less water when wearing a full face mask instead of an ordinary diving mask and even less when wearing a diving helmet. The latter two are therefore recommended when diving in fecally contaminated water.

**Figure 2:**
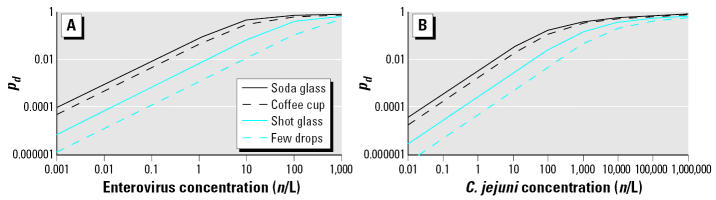
Risk of infection per dive, *p_d_*, with enteroviruses (*A*) and *C. jejuni* (*B*) as a function of pathogen concentration (*n*/L) in the diving water and the volume of swallowed water per dive.

For occupational divers and sport divers wearing an ordinary diving mask, the risk of infection with *C. jejuni* was estimated to be near 1% per dive, and the risk of infection with enteroviruses was near 0.1% per dive. The annual infection risks were one order of magnitude higher, dependent on pathogen concentration and the number of dives. These relatively high estimates indicate that the risk of infection from diving may be a significant health problem. Figure 2 shows the risk of infection with enteroviruses and *Campylobacter* species as a function of the concentration of these pathogens and the volume of swallowed water per dive. With these Figure 2 and the data from Table 1 (volume description and milliliters) and Table 2 (concentration ranges of pathogens in different types of water), one can estimate the risk associated with a certain swallowed volume of water and a particular level of contamination of that water. In the Netherlands, a maximum risk of infection of 10^−4^ per person per annum is applied as the Dutch legal standard for drinking water (Staatsblad 2001). A risk of infection of 10^−4^ is exceeded in water with > 0.001–0.1 enteroviruses per liter or > 0.03–3 *C. jejuni* per liter, dependent on swallowing a few drops up to a soda glass of water. A risk of infection 10^−2^ is notable from an epidemiologic point of view (Craun et al. 1996). This risk of infection is exceeded in water with > 0.1–10 enteroviruses per liter or > 3–300 *C. jejuni* per liter, dependent on the volume of swallowed water.

From the data on age, body length, and weight of the occupational and sport divers and the fact that a physically active life is inherent to divers, we may conclude that they represent a group of healthy adults. Nevertheless, only 20% of both the occupational and sport divers reported having none of the health complaints. Although the reported health complaints may not have been caused by diving activities, the high incidence of reported health complaints suggests that divers are subject to a higher risk of infection with waterborne pathogens due to diving.

De Wit et al. (2001a) estimated gastroenteritis incidence for adult persons in the Netherlands to be 283/1,000 person-years. Gastroenteritis was defined as three or more loose stools in 24 hr, or diarrhea with two or more additional symptoms, or includes vomiting with two or more additional symptoms, such as abdominal pain and cramps, nausea, fever, blood in the stool, mucus in the stool, diarrhea, or vomiting. An episode has to be preceded by a symptom-free period of 2 weeks. It also includes vomiting three or more times in 24 hr. In the present study, it was not possible to apply this case definition of gastroenteritis because no information was collected on concurrency of symptoms. Nevertheless, the reported incidences of nausea and vomiting are in reasonable agreement with the gastroenteritis incidence of 283 per ≥1,000 person-years. However, the reported incidence of diarrhea for sport divers of 400 per 1,000 person-years seems to be high and that for occupational divers (610 per 1,000 person-years) even higher. In 2002 and 2003, the incidence rate of gastroenteritis in general practices in the Netherlands for men 15–64 years of age was 67 and 74 per 10,000 person-years, respectively (Bartelds 2005). Incidence rates of the sport divers for nausea, vomiting, and diarrhea in general practices were 62, 41, and 125 per 10,000 person-years, respectively. This is similar to the data of Bartelds (2005). Therefore, our data suggest that the incidence of gastroenteritis-like symptoms of divers is similar to that of the Dutch population (excluding young children and the elderly). The incidence of ear complaints is high (500 and 530 per 1,000 person-years for sport and occupational divers, respectively).

The present study has given insight into the high risks of infection that divers may experience in fecally contaminated water. The exposure data collected in this study can also be of use for risk assessments due to the exposure to toxic and carcinogenic agents (Maibach 1975; Richter et al. 2003). Divers should be made aware of these risks, and protective actions such as wearing the appropriate diving gear should be taken. Legal frameworks that aim toward this are provided by the Dutch Working Conditions Decree (2000), which, among other things, requires a risk assessment and evaluation of a diving location, EU Directive 90/679/EEC (1990) on the protection of workers from risks related to exposure to biologic agents at work, and the proposed new EU bathing water directive COD 2002/0254 (2002), which is aimed at better informing water recreants on the risks of bathing by means of bathing water profiles.

## Figures and Tables

**Table 1:** Number (%) of divers who swallowed the specified volume of water per dive [shown as range (average)].

	Water volume (mL)					
	None 0 (0)	Few drops 0.5–5 (2.75)	Shot glass 20–30 (25)	Coffee cup 80–120 (100)	Soda glass 170–210 (190)	Total divers
Occupational divers in 2002						
Open sea	5 (25)	9 (45)	6 (30)	0 (0)	0 (0)	20
Coastal water, usd < 1 km	4 (50)	1 (13)	3 (38)	0 (0)	0 (0)	8
Coastal water, usd > 1 km	2 (29)	3 (43)	2 (29)	0 (0)	0 (0)	7
Coastal water, usd unknown	5 (18)	8 (29)	4 (14)	1 (3.6)	0 (0)	18
Fresh water, usd < 1 km	4 (33)	6 (50)	2 (17)	0 (0)	0 (0)	12
Fresh water, usd > 1 km	4 (33)	6 (50)	2 (17)	0 (0)	0 (0)	12
Fresh water, no usd	7 (58)	3 (25)	2 (17)	0 (0)	0 (0)	12
Fresh water, usd unknown	11 (44)	9 (36)	5 (20)	0 (0)	0 (0)	25
Sport divers in 2003 wearing an ordinary diving mask						
Open sea	130 (46)	39 (14)	102 (36)	9 (3.2)	0 (0)	280
Coastal water	57 (14)	79 (19)	262 (64)	10 (2.4)	2 (0.49)	410
Fresh recreational water	35 (8.3)	110 (26)	255 (61)	20 (6.8)	1 (0.24)	421
Canals and rivers	126 (63)	13 (6.5)	59 (30)	2 (1.0)	0 (0)	200
City canals	130 (75)	8 (4.6)	33 (19)	2 (1.1)	0 (0)	173
Swimming pools	47 (14)	91 (28)	154 (47)	28 (8.6)	6 (1.8)	326
Sport divers in 2003 wearing a full face mask						
Open sea	27 (84)	5 (16)	0 (0)	0 (0)	0 (0)	32
Coastal water	25 (96)	0 (0)	1 (4.0)	0 (0)	0 (0)	26
Fresh recreational water	27 (96)	0 (0)	1 (4.0)	0 (0)	0 (0)	28
Canals and rivers	24 (83)	5 (17)	0 (0)	0 (0)	0 (0)	29
City canals	24 (89)	3 (10)	0 (0)	0 (0)	0 (0)	27
Swimming pools	23 (74)	0 (0)	5 (16)	2 (6.5)	1 (3.2)	31

usd, upstream sewage discharge.

**Table 2:** Concentration ranges of *C. jejuni* and enteroviruses.

Literature data		Applied concentration		
			Concentration (*n*/L)	
Type of water	Concentration range (*n*/L)	Type of water	Range	Arithmetic mean
*C. jejuni*^a^				
Coastal water	10^2^–10^4^	Open sea and all surface water with usd > 1 km or unknown		
Slaughterhouse wastewater	10^2^–10^6^		10^−2^–10^4^	3,600
Sewage sludge	10^4^–10^5^	All surface water, usd < 1 km	1–10^4^	4,900
Raw wastewater	10^−2^–10^4^			
Treated wastewater	1–10^4^			
Rivers and streams	10^−2^–10^4^			
Lakes, ponds, reservoirs	10^−2^–10^4^			
Enteroviruses^b^				
Coastal water	10^−3^–1	Open sea	10^−5^–10	0.36
Raw wastewater	1–10^3^	All surface water, usd < 1 km	10^−2^–10	0.78
Treated wastewater	10^−2^–10^2^	All surface water, usd unknown	10^−3^–10	0.44
Rivers and streams	10^−2^–10	All surface water, usd > 1 km	10^−3^–1	0.078
Lakes, ponds, reservoirs	10^−3^–1			
Recreational waters	10^−3^–1			

usd, upstream sewage discharge.

a Data from Havelaar et al. (2001) and Schijven (2003).

b Data from Havelaar et al. (1993), Hoogenboezem et al. (2000), Lodder and de Roda Husman (2005), and Schijven (2003).

**Table 3:** Arithmetic mean (maximum) number of dives per diver and volume of swallowed water (mL) per dive.

Divers, location	Percent of divers	No. of dives per diver	Swallowed water per dive (mL)
Occupational divers in 2002 (*n* = 35)			
Open sea	57	24 (151)	8.7 (25)
Coastal water, usd < 1 km	23	3.2 (36)	9.7 (25)
Coastal water, usd > 1 km	20	1.8 (16)	8.3 (25)
Coastal water, usd unknown	51	16 (200)	12 (100)
Open sea and all coastal water combined			9.8 (100)
Fresh water, usd < 1 km	37	8.3 (76)	5.5 (25)
Fresh water, usd > 1 km	37	16 (200)	5.5 (25)
Fresh water, no usd	37	16 (200)	4.8 (25)
Fresh water, usd unknown	77	45 (200)	6.0 (25)
All fresh waters combined			5.7 (25)
Sport divers in 2003 (*n* = 482) wearing an ordinary diving mask			
Open sea	26	2.1 (120)	7.7 (100)
Coastal water	78	14 (114)	9.9 (190)
Open sea and coastal water combined			9.0 (190)
Fresh recreational water	85	22 (159)	13 (190)
Canals and rivers	11	0.65 (62)	3.4 (100)
City canals	1.5	0.031 (4)	2.8 (100)
Canals, rivers, and city canals combined			3.2 (100)
Swimming pools	65	17 (134)	20 (190)
Sport divers in 2003 (*n* = 482) wearing a full face mask			
Open sea	0.21	0.012 (6)	0.43 (2.8)
Coastal water	1.0	0.10 (34)	1.3 (15)
Fresh recreational water	2.7	0.44 (80)	1.3 (15)
Canals and rivers	1.2	0.098 (13)	0.47 (2.8)
City canals	0.41	0.010 (3)	0.31 (2.8)
All surface waters combined			0.81 (25)
Swimming pools	2.3	0.21 (40)	13 (190)

usd, upstream sewage discharge. All minimum values were zero.

**Table 4:** Arithmetic mean percent (97.5th percentile) risk of infection per dive, *p_d_*, and per year, *p_y_*

	*C. jejuni*		Enteroviruses	
Divers, location	*p_d_* (%)	*p_y_* (%)	*p_d_* (%)	*p_y_* (%)
Occupational divers in 2002 (*n* = 35)				
Open sea	1.4 (15)	23 (96)	0.12 (0.72)	1.8 (9.3)
Coastal water, SD < 1 km	2.8 (22)	6.4 (50)	0.32 (2.5)	1.2 (15)
Coastal water, SD > 1 km	1.2 (13)	2.0 (30)	0.033 (0.27)	0.030 (0.43)
Coastal water, SD unknown	1.3 (14)	14 (97)	0.19 (1.4)	2.4 (23)
Fresh water, SD < 1 km	1.8 (17)	13 (88)	0.2 (1.6)	2.5 (24)
Fresh water, SD > 1 km	0.88 (9.8)	10 (72)	0.02 (0.16)	0.35 (4.5)
Fresh water, no SD	0.88 (10)	12 (76)	0.019 (0.16)	0.26 (1.8)
Fresh water, SD unknown	0.9 (9.9)	29 (84)	0.13 (0.94)	6.4 (53)
Sport divers in 2003 (*n* = 482) wearing an ordinary diving mask				
Open sea	1.1 (11)	2.1 (27)	0.12 (0.55)	0.27 (1.8)
Coastal water	1.1 (11)	13 (58)	0.18 (1.4)	2.4 (17)
Fresh recreational water	1.5 (15)	25 (80)	0.040 (0.35)	1.0 (4.1)
Canals and rivers	0.44 (4.0)	0.17 (1.4)	0.063 (0.44)	0.030 (0.12)
City canals	0.44 (4.1)	0.00032	0.079 (0.44)	0.0039
Sport divers in 2003 (*n* = 482) wearing a full face mask				
Open sea	0.18 (1.1)	0.00020	0.014 (0.032)	0.000032
Coastal water	0.16 (1.2)	0.0084	0.018 (0.12)	0.0021
Fresh recreational water	0.15 (1.1)	0.14	0.0031 (0.026)	0.0023
Canals and rivers	0.17 (1.2)	0.0077	0.017 (0.12)	0.0049
City canals	0.15 (0.91)	0.00011	0.017 (0.12)	0.00020

usd, upstream sewage discharge. All 2.5th percentiles values were zero; values for 97.5th percentiles that equaled zero were omitted.

## References

[b1-ehp0114-000712] Ahlén C, Mandal LH, Iversen OJ (2003). An infield demonstration of the true relationship between skin infections and their sources in occupational diving systems in the North Sea. Ann Occup Hyg.

[b2-ehp0114-000712] Alcock SR (1977). Acute otitis externa in divers working in the North Sea: a microbiological survey of seven saturation dives. J Hyg.

[b3-ehp0114-000712] Bartelds AIM 2005. Continue Morbiditeits Registratie Peilstations Nederland 2004 [in Dutch]. Utrecht, the Netherlands:NIVEL.

[b4-ehp0114-000712] Craun G, Dufour A, Eisenberg J, Foran J, Gauntt C, Gerba C (1996). Conceptual framework to assess the risks of human disease following exposure to pathogens. Risk Anal.

[b5-ehp0114-000712] de Roda Husman AM (2001). Human viruses in H_2_O [in Dutch]. H2O.

[b6-ehp0114-000712] Dewailly E, Poirier C, Meyer FM (1986). Health hazards associated with windsurfing on polluted water. Am J Public Health.

[b7-ehp0114-000712] De Wit MAS, Koopmans MPG, Kortbeek LM, Wannet WJB, Vinjé J, van Leusden F (2001a). Sensor, a population-based cohort study on gastroenteritis in the Netherlands, incidence and aetiology. Am J Epidemiol.

[b8-ehp0114-000712] De Wit MAS, Kortbeek LM, Koopmans MPG, de Jager CJ, Wannet WJB (2001b). A comparison of gastroenteritis in a general practice-based study and a community-based study. Epidemiol Infect.

[b9-ehp0114-000712] Dutch Working Conditions Decree 2000. Bulletin of Acts, Orders and Decrees, 327.

[b10-ehp0114-000712] EU (European Union) 1976. Directive 76/160/EEC. Concerning the Quality of Bathing Water.

[b11-ehp0114-000712] EU (European Union) 1990. Directive 90/679/EEC. On the Protection of Workers from Risks Related to Exposure to Biological Agents at Work.

[b12-ehp0114-000712] EU (European Union) 2002. COD 2002/0254. Proposal for a Directive of the European Parliament and of the Council Concerning the Quality of Bathing Waters. COM/2002/0581 final. Available: http://europa.eu.int/eur-lex/en/com/pdf/2002/com2002_0581en01.pdf [accessed 15 March 2006].

[b13-ehp0114-000712] Garin D, Fuchs F, Crance JM, Rouby Y, Chapalain JC, Lamarque D (1994). Exposure to enteroviruses and hepatitis A virus among divers in environmental waters in France, first biological and serological survey of a controlled cohort. Epidemiol Infect.

[b14-ehp0114-000712] Havelaar AH ed. 2001. Campylobacteriose in Nederland-Risico’s en interventiemogelijkheden [in Dutch] RIVM report 25091100. Bilthoven, the Netherlands:National Institute for Public Health and the Environment.

[b15-ehp0114-000712] Havelaar AH, Olphen van M, Drost YC (1993). F-specific RNA bacteriophages are adequate model organisms for enteric viruses in fresh water. Appl Environ Microbiol.

[b16-ehp0114-000712] Hoogenboezem W Ketelaars HAM Medema GJ Rijs G Schijven JF 2000. Cryptosporidium and Giardia: Presence in Sewage, Manure and Surface Water with Bathing- and Drinking Water Function. RIWA/RIVM/RIZA-report. Amsterdam:RIWA, Association of River Waterworks.

[b17-ehp0114-000712] Kay D, Fleisher JM, Salmon RL, Jones F, Wyer MD, Godfree AF (1994). Predicting likelihood of gastroenteritis from sea bathing: results from randomized exposure. Lancet.

[b18-ehp0114-000712] Leenen EJTM, de Roda Husman AM (2004). Health complaints associated with recreation in surface water in the summers of 2000, 2001, 2002 [in Dutch]. Infect Bull.

[b19-ehp0114-000712] Lodder WJ, de Roda Husman AM (2005). Presence of noroviruses and other enteric viruses in sewage and surface waters in the Netherlands. Appl Environ Microbiol.

[b20-ehp0114-000712] Losonsky GA, Hasan JAK, Huq A, Kaintuck S, Colwell RR (1994). Serum antibody responses of divers to waterborne pathogens. Clin Diagn Lab Immunol.

[b21-ehp0114-000712] Maibach H (1975). Scuba diver facial dermatitis: allergic contact dermatitis to *N*-isoporpyl-*N*-phenylparaphylenediamine. Contact Dermatitis.

[b22-ehp0114-000712] NOB 2006. Nederlandse Onderwatersport Bond Homepage. Utrecht, the Netherlands: Nederlandse Onderwatersport Bond. Available: http://www.nob-nl.nl/ [accessed 15 March 2006].

[b23-ehp0114-000712] Richter ED, Friedman LS, Tamir Y, Berman T, Levy O, Westin JB (2003). Cancer risks in naval divers with multiple exposures to carcinogens. Environ Health Perspect.

[b24-ehp0114-000712] RIVM 2006. National Institute for Public Health and the Environment Homepage. Bilthoven, the Netherlands:National Institute for Public Health and the Environment. Available: http://www.rivm.nl/en/ [accessed 15 March 2006].

[b25-ehp0114-000712] Schets FM, de Roda Husman AM (2004). Health complaints associated with recreation in surface water in the summer of 2003 [in Dutch]. Infect Bull.

[b26-ehp0114-000712] Schijven JF (2003). Assessment of the risk of infection with *Campylobacter* via water [in Dutch]. H2O.

[b27-ehp0114-000712] Schijven JF, Bradford SA, Yang S (2004). Release of *Cryptosporidium* and *Giardia* from dairy cattle manure: physical factors. J Environ Qual.

[b28-ehp0114-000712] Staatsblad Waterleidingbesluit 2001. Staatsblad nr 31 [in Dutch].

[b29-ehp0114-000712] Statistics Netherlands Statline. Available: http://statline.cbs.nl/StatWeb/Start.asp?lp=Search/Search&LA=EN&DM=SLEN [accessed 20 March 2005].

[b30-ehp0114-000712] Teunis PFM, Havelaar AH (2000). The beta Poisson dose response model is not a single-hit model. Risk Anal.

[b31-ehp0114-000712] Van Asperen IA, Medema G, Borgdorff MW, Sprenger MJW, Havelaar AH (1998). Risk of gastroenteritis among triathletes in relation to faecal pollution of fresh waters. Int J Epidemiol.

[b32-ehp0114-000712] Van Asperen IA, Rover de CM, Schijven JF, Oetomo SB, Schellekens JFP, Leeuwen van NJ (1995). Risk of otititis externa after swimming in recreational fresh water lakes containing *Pseudomonas aeruginosa*. BMJ.

[b33-ehp0114-000712] Van den Berg H, Lodder W, van der Poel W, Vennema H, de Roda Husman AM (2005). Genetic diversity of noroviruses in raw and treated sewage water. Res Microbiol.

[b34-ehp0114-000712] Wiedenmann A Krüger P Dietz K López-Pila JM Szewzyk R Botzenhart K 2006. A randomized controlled trial assessing infectious disease risks from bathing in fresh recreational waters in relation to the concentration of *Escherichia coli*, intestinal enterococci, *Clostridium perfringens*, and somatic coliphages. Environ Health Perspect 114228–236; 10.1289/ehp.8115 [Online 29 September 2005]. 10.1289/ehp.8115 PMC1367836 16451859

